# Detection of *Burkholderia thailandensis* in Soil Samples, Suriname

**DOI:** 10.3201/eid3110.251114

**Published:** 2025-10

**Authors:** Jelmer Savelkoel, Rosalie Zimmermann, Ansmarie Ngu Chin Tjon, Tsira Dzebisasjvili, Maren Lanzl, Sébastien Matamoros, Terrence Mawie, Lycke Woittiez, Stephen Vreden, Emma Birnie, W. Joost Wiersinga

**Affiliations:** Amsterdam UMC location University of Amsterdam, Amsterdam, the Netherlands (J. Savelkoel, R. Zimmermann, S. Matamoros, E. Birnie, W.J. Wiersinga); Centre for Agricultural Research in Suriname, Paramaribo, Suriname (A. Ngu Chin Tjon); National Institute for Public Health and the Environment, Bilthoven, the Netherlands (T. Dzebisasjvili, M. Lanzl); Academic Hospital Paramaribo, Paramaribo (T. Mawie, L. Woittiez); Foundation for the Advancement of Scientific Research in Suriname, Paramaribo (S. Vreden)

**Keywords:** Burkholderia thailandensis, Burkholderia pseudomallei, Suriname, epidemiology, environmental sampling, bacteria

## Abstract

Melioidosis, caused by the highly lethal pathogen *Burkholderia pseudomallei*, is emerging in North and South America. We studied soil samples in Suriname to determine endemicity of *Burkholderia* species. *B. thailandensis* was isolated, but *B. pseudomallei* was not. A multidisciplinary approach could establish clinical and ecologic distribution of both *Burholderia* species in Suriname.

The epidemiology of the environmental gram-negative bacteria *Burkholderia pseudomallei*, the highly lethal pathogen causing melioidosis, and less lethal *B. thailandensis* is changing because of climatic conditions (*1*,*2*). During 2019–2022, *B. pseudomallei* and *B. thailandensis*, both part of the *B. pseudomallei* complex, were isolated from the environment in the continental United States (*2*,*3*). A large set of whole-genome sequence data indicates that *B. pseudomallei* could have been introduced from Africa to North and South America between 1650 and 1850 through the slave trade (*4*), and that might also be true for *B. thailandensis*. 

Suriname, a tropical country in South America with an ethnically diverse population of <1 million inhabitants, could be a historic port of entry for *Burkholderia* species, especially considering *B. pseudomallei* is established in neighboring countries Brazil and (presumably) French Guiana (*5*,*6*). However, neither *Burkholderia* species has yet been identified in Suriname. We performed an environmental sampling study to determine whether *B. pseudomallei* or *B. thailandensis* could be isolated from soil samples in Suriname.

We collected soil samples in Suriname during the major wet season in May–July 2023 (https://climateknowledgeportal.worldbank.org/country/suriname/climate-data-historical) by using methods from the consensus guidelines for isolation of *B. pseudomallei* and our previous work in Nigeria (*7*,*8*). We selected 4 sites in the northern districts Paramaribo, Commewijne, and Nickerie and collected 100 samples at each site at a depth of 60–80 cm (Table). We determined the soil acidity and alkalinity and the salinity of air-dried mixed soil samples by measuring the pH and electrical conductivity. Next, we cultured 10 g of soil by using selective broth and agar and inspected agar plates daily for suspect isolates. We subcultured morphologically suspect isolates onto blood agar plates and screened for oxidase positivity by using oxidase strips (Sigma-Aldrich, https://www.sigmaaldrich.com) and antimicrobial resistance testing by using disks (Thermo Fisher Scientific, https://www.thermofisher.com) to assess susceptibility to amoxicillin/clavulanic acid at 30 μg (defined by the presence of an inhibition zone) and resistance to colistin at 10 μg and gentamicin at 10 μg (defined by the absence of an inhibition zone), considered an adjustment of the standard operating procedure of the consensus methods (*7*,*9*). Next, we subjected isolates matching those criteria to a real-time multiplex PCR on the LightCycler 480 II (Roche, https://www.lifescience.roche.com) and subsequent presumed *B. thailandensis* isolates were confirmed by using whole-genome sequencing on the NextSeq 500 platform (Illumina, https://www.illumina.com) (Appendix). We uploaded whole-genome sequences of the *B. thailandensis* isolates to the European Nucleotide Archive database (https://www.ebi.ac.uk/ena; project no. PRJEB79520, sample accession nos. ERS20924713–6).

**Table:** Site and sample characteristics for detection of *Burkholderia thailandensis* in soil samples, Suriname*

Site	Region	District	Location	Site characteristics	pH	Electrical conductivity, μS/cm	No. positive samples
A	Northern	Paramaribo	Centre for Agricultural Research in Suriname	Inceptisol soil type; brackish field from which former crops included sem bean, banana, cassava, peanut, corn, and soybeans	7.30	769	0
B	Northern	Commewijne	Plantation Rust en Werk	Inceptisol soil type; field with cattle	4.42	1,313	0
C	Northwest	Nickerie	Europolder	Inceptisol soil type; brackish rice field	7.66	908	0
D	Northwest	Nickerie	Middenstandspolder	Inceptisol soil type; brackish rice field	6.83	1,097	4

*At each site, 100 soil samples were collected at a depth of 60–80 cm.

We collected 400 soil samples across 4 different sites and identified 4 *B. thailandensis* isolates at 1 site in the Nickerie district (Table). However, we did not detect *B. pseudomallei*. Sequence data revealed that the *B. thailandensis* isolates were phylogenetically distinct because they differed by 2,965–3,226 single-nucleotide polymorphisms from each other, suggesting multiple introductory events. We downloaded paired-end sequences of other *B. thailandensis* isolates with data on country and source from the European Nucleotide Archive database, including isolates identified in the continental United States and Nigeria (*2*,*8*), and constructed a phylogenetic tree (Figure). The isolates from Suriname clustered in the same clade with the isolates from the United States and Nigeria, suggesting the Suriname isolates could have descended from Africa.

**Figure:**
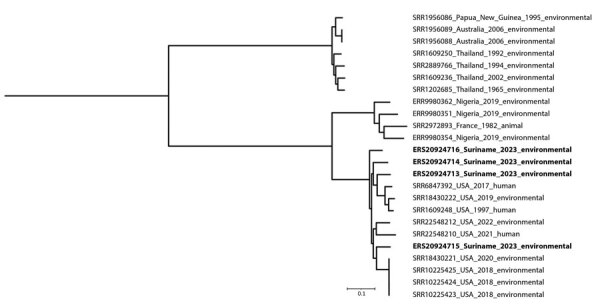
Phylogeny of *Burkholderia thailandensis* detected in soil samples, Suriname. Maximum-likelihood tree generated by FastTree (http://www.microbesonline.org/fasttree) on the basis of core single-nucleotide polymorphism distance and visualized with iTOL (https://itol.embl.de). Bold indicates isolates from this study. Additional global genomes, all retrieved from the European Nucleotide Archive database (https://www.ebi.ac.uk/ena), are indicated by accession number, country name, year of isolation, and isolate source. Scale bar indicates nucleotide substitutions per site.

We might have underestimated the true distribution of *B. thailandensis* and cannot rule out the presence of *B. pseudomallei* because of the small sample size and reliance on outdated soil-focused consensus guidelines based on soil sampling in highly endemic areas (*7*). Furthermore, additional water and air sampling might have yielded a higher success rate in determining endemicity for *B. pseudomallei* but are not yet an integral part of the adopted guidelines (*7*).

A strength of our study is performing an environmental sampling study in different districts in the northern parts of Suriname, where the population and agricultural practices are dense and interaction between humans and soil is highly likely. However, we did not find *B. pseudomallei*, which might reflect a low soil abundance. In line with this finding, a previous study of traveler-associated melioidosis cases in the Netherlands did not identify any cases that originated in Suriname (*10*), despite frequent family visits between Suriname and the Netherlands. Our phylogenetic results could support the theory that *B. thailandensis* was introduced to the Americas through slave trade, similar to the continental introduction of *B. pseudomallei*, suggested by others on the basis of sequence data of 469 isolates (*4*).

In conclusion, we detected *B. thailandensis* in soil samples from Suriname. Although we did not detect *B. pseudomallei*, a multidisciplinary approach could clarify the clinical and ecologic distribution of both *Burholderia *species in Suriname.

Appendix. Additional information on detection of *Burkholderia thailandensis* in soil samples, Suriname.
