# Supplementary material for: Detection of Burkholderia thailandensis in Soil Samples, Suriname
Source: Emerg Infect Dis. 2025 Oct;31(10):2057–9. doi: 10.3201/eid3110.251114 (PMC12483106; doi:10.3201/eid3110.251114)
Supplement: Supplementary file 1 — Appendix. Additional information on detection of Burkholderia thailandensis in soil samples, Suriname. [file 25-1114-Techapp-s1.pdf]

Article DOI: https://doi.org/10.3201/3110.251114
EID cannot ensure accessibility for supplementary materials supplied by authors. Readers who have difficulty accessing supplementary content should contact the authors for assistance.

Detection of *Burkholderia thailandensis* in Soil Samples, Suriname

Polymerase Chain Reaction and Whole-Genome Sequencing

Polymerase chain reaction and whole-genome sequencing were performed as previously described and sequences were processed by using the latest available software versions including kSNP4 v4.1 (*1*,*2*). We cleaned one of our *Mycobacterium tuberculosis* contaminated *B. thailandensis* sequences (ERS20924713) with a publicly available tool and NC_000962.3 as reference sequence, followed by extraction of the unmapped *B. thailandensis* reads with SAMtools v1.17 (*3*).

References

1. Savelkoel J, Oladele RO, Ojide CK, Peters RF, Notermans DW, Makinwa JO, et al. Presence of *Burkholderia pseudomallei* in soil, Nigeria, 2019. Emerg Infect Dis. 2023;29:1073–5. PubMed https://doi.org/10.3201/eid2905.221138

2. Hall BG, Nisbet J. Building phylogenetic trees from genome sequences with kSNP4. Mol Biol Evol. 2023;40:msad235. PubMed https://doi.org/10.1093/molbev/msad235

3. Danecek P, Bonfield JK, Liddle J, Marshall J, Ohan V, Pollard MO, et al. Twelve years of SAMtools and BCFtools. Gigascience. 2021;10:giab008. PubMed https://doi.org/10.1093/gigascience/giab008
